# Novel Basic Science Insights to Improve the Management of Heart Failure: Review of the Working Group on Cellular and Molecular Biology of the Heart of the Italian Society of Cardiology

**DOI:** 10.3390/ijms21041192

**Published:** 2020-02-11

**Authors:** Pietro Ameri, Gabriele Giacomo Schiattarella, Lia Crotti, Margherita Torchio, Edoardo Bertero, Daniele Rodolico, Maurizio Forte, Vittoria Di Mauro, Roberta Paolillo, Cristina Chimenti, Daniele Torella, Daniele Catalucci, Sebastiano Sciarretta, Cristina Basso, Ciro Indolfi, Cinzia Perrino

**Affiliations:** 1 IRCCS Ospedale Policlinico San Martino—IRCCS Italian Cardiovascular Network & Department of Internal Medicine, University of Genova, 16132 Genova, Italy; pietroameri@unige.it; 2 Department of Internal Medicine (Cardiology), University of Texas Southwestern Medical Center, Dallas, TX 75206, USA; Gabriele.Schiattarella@UTSouthwestern.edu; 3 Istituto Auxologico Italiano, IRCCS, Department of Cardiovascular, Neural and Metabolic Sciences, San Luca Hospital, 20149 Milan, Italy; l.crotti@auxologico.it; 4 Department of Medicine and Surgery, University of Milano-Bicocca, 20126 Milan, Italy; 5 Istituto Auxologico Italiano, IRCCS, Istituto Auxologico Italiano, Center for Cardiac Arrhythmias of Genetic Origin, and Laboratory of Cardiovascular Genetics, 20095 Milan, Italy; m.torchio@auxologico.it; 6 Comprehensive Heart Failure Center (CHFC), University Clinic Würzburg, 97078 Würzburg, Germany; edo.bertero@gmail.com; 7 Agostino Gemelli Medical School, Catholic University of the Sacred Heart, 00168 Rome, Italy; daniele.rodolico01@icatt.it; 8 Department of AngioCardioNeurology, IRCCS Neuromed, 86077 Pozzili, Italy; maur.forte@gmail.com (M.F.); sebastiano.sciarretta@uniroma1.it (S.S.); 9 National Research Council (CNR) Institute of Genetics & Biomedical Research, Milan Unit, 20138 Milan, Italy; Vittoria.Di_Mauro@humanitasresearch.it (V.D.M.); daniele.catalucci@cnr.it (D.C.); 10 Humanitas Clinical and Research Hospital, 20090 Rozzano (MI), Italy; 11 Department of Advanced Biomedical Sciences, Federico II University, 80131 Naples, Italy; robe.paolillo@gmail.com; 12 Department of Cardiovascular, Respiratory, Nephrologic, and Geriatric Sciences, Sapienza University of Rome, 00100 Rome, Italy; cristinachimenti@libero.it; 13 Molecular and Cellular Cardiology Laboratory, Department of Experimental and Clinical Medicine, Magna Graecia University, 88100 Catanzaro, Italy; dtorella@unicz.it; 14 Department of Medical and Surgical Sciences and Biotechnologies, Sapienza University of Rome, 04100 Latina, Italy; 15 Cardiovascular Pathology, Department of Cardiac, Thoracic, Vascular Sciences and Public Health, University of Padua, 35121 Padua, Italy; cristina.basso@unipd.it; 16 Division of Cardiology, Department of Medical and Surgical Sciences & Center of Cardiovascular Research, Magna Graecia University, 88100 Catanzaro, Italy; indolfi@unicz.it; 17 URT-CNR, Magna Graecia University, 88100 Catanzaro, Italy

**Keywords:** heart failure, basic, translational, research, mechanisms

## Abstract

Despite important advances in diagnosis and treatment, heart failure (HF) remains a syndrome with substantial morbidity and dismal prognosis. Although implementation and optimization of existing technologies and drugs may lead to better management of HF, new or alternative strategies are desirable. In this regard, basic science is expected to give fundamental inputs, by expanding the knowledge of the pathways underlying HF development and progression, identifying approaches that may improve HF detection and prognostic stratification, and finding novel treatments. Here, we discuss recent basic science insights that encompass major areas of translational research in HF and have high potential clinical impact.

## 1. Introduction

Heart failure (HF) is a highly prevalent condition with devastating impact on patients’ and caregivers’ lives, enormous health care costs, and dismal prognosis. Several therapies are now available for HF with reduced left ventricular ejection fraction (HFrEF), but in the majority of patients these treatments only slow the natural history of the syndrome, with functional and structural recovery being partial even when clinical stability is attained [1]. In contrast, HF with preserved LV ejection fraction (HFpEF) remains orphan of specific treatment [2]. The burden of HF is compounded by a steadily growing prevalence [1]. This trend is primarily due to ageing of the general population, since age is a risk factor for HF in itself, and major advances in the management of acute cardiac diseases, such as myocardial infarction (MI), which have led to increased survival in the short term, but have also made HF-predisposing long-term sequelae common [3]. Therefore, prevention of HF onset or progression and improvement of treatment once HF has developed represent major objectives of contemporary medicine. 

Basic science is expected to give fundamental inputs to accomplish these goals, by collecting evidence that may lay the foundations for or allow a better understanding of novel diagnostic, prognostic or therapeutic tools [4]. For instance, the discovery of the I_f_ pacemaker current has led to the development of ivabradine [5], which in turn has become a medication for HFrEF [6]. More recently, sodium-glucose transport protein 2 (SGLT2) inhibitors have been proved to reduce HF hospitalizations and cardiovascular mortality, and the underlying mechanisms are still being revealed [7,8]. 

**Figure 1:**
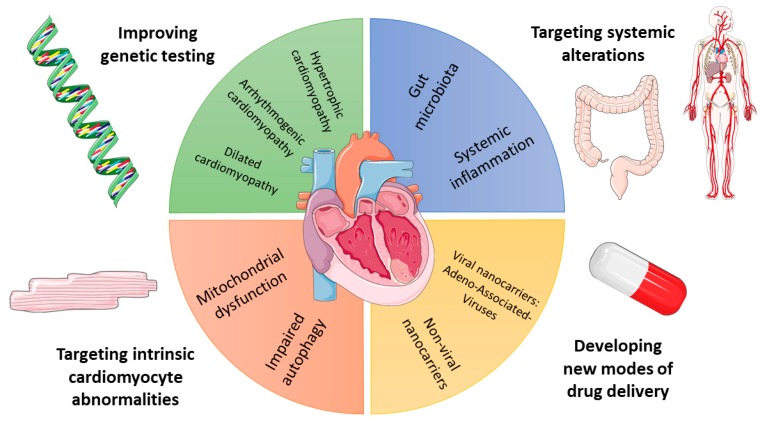
Areas of basic science research into heart failure covered by this review.

Here, we discuss recent basic science insights with potential clinical impact on HF. In the effort of providing a comprehensive overview, the main domains of translational research in HF are covered: genetics, cardiomyocyte-intrinsic defects, systemic alterations bidirectionally linked to HF, and therapeutic strategies (Figure 1).

## 2. Depicting the Genetic Background of Inherited HF 

The use of next-generation sequencing (NGS) has made possible to identify several gene mutations or variants that are associated with inherited cardiomyopathies eventually leading to HF. Genetically determined cardiomyopathies most often associated to HF are hypertrophic (HCM), arrhythmogenic (ACM) and dilated (DCM) cardiomyopathies. When one of these inherited cardiomyopathies is diagnosed, molecular screening is a class I (HCM) or IIa (familial DCM and ACM) indication in the proband, while in family members it is always a class I indication whenever the disease-causing mutation is identified in the proband [9].

In patients affected by HCM a disease-causing mutation is identified in around 40-60% of the cases, and the yield of genetic testing is higher in individuals who have a family history of HCM [10,11]. Sarcomeric genes more frequently implicated are β-myosin heavy chain 7 (*MYH7*) and myosin binding protein C (*MYBPC3*), accounting for approximately 80% of all positive cases [12,13]. Other seven sarcomeric genes are less frequently implicated, namely those encoding cardiac troponin I (*TNNI3*), α-tropomyosin (*TPM1*), myosin regulatory light chain (*MYL2*), myosin essential light chain (*MYL3*), cardiac α-actin (*ACTC1*), and cysteine and glycine-rich protein 3 (*CSRP3*) [14,15]. Non-sarcomeric genes associated with HCM have been reported in a small minority of patients, including genes encoding Z-disk, sarcoplasmic reticulum or plasma membrane proteins [15]. However, variants in these genes occur less commonly and studies providing solid evidence for their implication in the disease are limited.

ACM is classically described as a disease of the desmosome, a multiprotein complex that forms cell-to-cell junctions and links intermediate filaments of adjacent cells. Mutations in five genes that encode desmosomal proteins (plakoglobin, desmoplakin, plakophilin-2, desmoglein-2, and desmocollin-2) have been found in ACM. However, the genetics of ACM may be more complex than monogenic heritability, with more than 1 mutation in the same gene (compound heterozygosity) or in different genes (digenic heterozygosity) being reported [16]. Fibro-fatty substitution, the histological hallmark of ACM, can involve the right ventricle (RV), the LV, or both. In fact, pure RV forms are rare, and some alterations of the LV can be demonstrated even when RV involvement predominates. Among mutations in the desmosomal genes associated with ACM, those in *DSP*, especially non-missense, are most often associated to LV involvement [17]. A number of non-desmosomal genes, including *TMEM43*, *PLN*, *SCN5A,* and *DES*, have also been associated with ACM, DCM, or overlap syndromes frequently complicated by malignant ventricular arrhythmias. Among these, the role of *SCN5A* in ACM is still not fully understood [18], while particular *PLN* mutations have been found to be associated with severe forms of ACM and DCM, characterized by early-onset severe HFrEF [19,20]. In the last few years, new genes have been implicated in the pathogenesis of ACM. By NGS, truncating mutations in *FLNC* have been identified not only in patients with DCM, but also in patients with left-dominant ACM, clearly highlighting the overlap between the two conditions [20]. More recently, mutations in the *CDH2* gene, encoding N-cadherin, have been identified through whole-exome sequencing in a three-generation family with ACM [21]. *N*-cadherin is a non-desmosomal protein of the intercalated disc, playing an important role in cell adhesion. This finding provided novel insight into the pathogenesis of ACM by changing the focus from the desmosome to the intercalated disc [21]. Indeed, along with *CTNNA3*, encoding alpha T-catenin [22], and *TJP1*, encoding zonula occludens 1 [23], *CDH2* is a major structural and functional component of intercalated discs, essential for cardiomyocyte connection.

The genetics of DCM is much more complex, with considerably more heterogeneity and a growing number of genes implicated. The yield of genetic testing for familial DCM has been estimated to be approximately 40% [24], whereas in isolated cases of DCM a disease-causing mutation is identified in 10–25% of patients. Variants in over 50 genes regulating different cellular components, such as the sarcomere, the nuclear envelope, the cytoskeleton and the sarcoplasmic reticulum, have been associated with familial DCM [25]. Furthermore, all HCM and ACM genes are usually included in diagnostic DCM panels, as a certain degree of genetic and phenotypic overlap can be present (Table 1). The most significant DCM genes, which should be always tested are: *TTN*, *LMNA*, *MYH7*, *TNNT2*, *BAG3*, *RBM20*, *TNNC1*, *TNNI3*, *TPM1*, *SCN5A*, *PLN,* and *FLNC* [26,27]; while there are a number of other minor genes that have been associated with DCM, but should be screened only in selected cases [28]. Among the major genes, *TTN* truncating mutations are the most common cause of DCM, occurring in ~25% of familial cases of DCM and in 18% of sporadic cases [29]. The *LMNA* gene is the second most commonly identified cause of DCM with a diagnostic yield of 5.5% [30]. *LMNA*-associated DCM has a distinct genotype–phenotype correlation, it is frequently associated with conduction abnormalities and the risk of sudden cardiac death (SCD) is definitely higher than in other forms of DCM [31]. In fact, risk stratification for SCD and the indication to implant a defibrillator in primary prevention are different for patients with a *LMNA* mutation [32]. *FLNC*, encoding filamin C, interacts with the dystrophin complex and causes a DCM phenotype when disrupted [32]. Truncating mutations in *FLNC* are associated with high risk of ventricular arrhythmias and SCD [32] and, therefore, the identification of a disease-causing mutation could have prognostic implications.

Finally, in the majority of patients with DCM genetics could play a role creating a favorable background, through the action of common genetic variants, on top of which triggering factors could act developing the disease. To identify such a genetic contribution to HF, some genome-wide association studies (GWAS) have been conducted in sporadic DCM cases and control subjects. In one such study, two common genetic variants were identified as significantly associated with DCM, one of which lied within the *BAG3* gene, a finding that also paved the way for the discovery of *BAG3* mutations in the familial form of DCM [33]. In another GWAS, class I and class II major histocompatibility complex heavy chain receptor genes were identified as susceptibility loci for DCM, highlighting the potential role that inflammation may play in the pathogenesis of the disease [34].

In summary, guided, but systematic implementation of genetic testing in clinical practice is expected to allow distinguishing precise cardiomyopathies within broad phenotypes, improving the effectiveness of the counselling for the patients and their relatives and better informing important clinical decisions, such as in particular implantation of a defibrillator. In parallel, GWAS may help understanding the pathogenesis of non-monogenic cardiomyopathies leading to HF.

## 3. Cardiomyocyte Abnormalities in HF: The Example of Autophagy

Many alterations characterize cardiomyocytes of the failing heart, and a comprehensive review of them is beyond the scopes of this work. This section will rather address autophagy, since this phenomenon is being increasingly investigated in HF and may be exploited to design novel therapies (Figure 1). 

Autophagy is an evolutionary conserved intracellular mechanism devoted to the removal of senescent or damaged cytoplasmic elements, which allows cells to adapt to stresses [35,36]. As a result, autophagy limits cell damage and death and plays a fundamental role in cardiac homeostasis and function [35,36,37,38]. A large body of evidence suggests that autophagy exerts both protective and detrimental effects in the heart, depending on the extent of activation and the type of stress [38]. In general, when autophagy is maintained within a physiological range, it preserves cardiac function and structure in response to stress. Conversely, low or massive levels of autophagy are deleterious for the heart [38]. Indeed, mice with inducible cardiac deletion of Atg5, a protein pivotal for autophagy activation, die prematurely because of cardiac dysfunction [37].

Three different forms of autophagy have been characterized: macro-autophagy, micro-autophagy, and chaperone-mediated autophagy (CMA) [35,36]. In macro-autophagy (hereafter called autophagy), cytoplasmic cargoes are sequestered by double membrane vesicles called autophagosomes and then delivered to lysosomes. This process may be specific, for instance, mitophagy is selective autophagy of diseased mitochondria [39,40]. Autophagy is stimulated during energy stress or nutrient deprivation allowing the supply of new metabolic substrates for ATP generation and sustaining cellular energy status [35]. Autophagy is also activated in response to mitochondrial damage, by sensing changes in mitochondrial potential membrane or low ATP levels [40]. Endoplasmic reticulum (ER) stress represents another condition that triggers autophagy in cardiomyocytes, especially when unfolded proteins cannot be removed by proteasomes [41]. Reactive oxygen species (ROS) also modulate autophagy during cellular stress. In most cases, autophagy acts as a pro-survival mechanism in the presence of elevated levels of ROS whereas in other cases ROS act as intracellular messengers to directly modulate autophagy [42,43]. Different signaling pathways acting as sensors of cell energy status modulate autophagy. The mammalian target of rapamycin (mTOR) is an atypical protein kinase which acts as a negative regulator of autophagy whereas 5’-AMP-activated kinase (AMPK) and glycogen synthase kinase-3 beta (GSK-3β) positively regulate autophagy [38,44]. Autophagy is also inhibited by Mst1, a protein kinase that triggers cell death [38]. Furthermore, transcription factor EB (TFEB) enhances the expression of genes involved in lysosomal biogenesis and Atg proteins, key molecules involved in the regulation of all the phases of autophagy process [45].

Cardiac autophagy progressively declines during aging. Overexpression of Atg5 was found to delay cardiac aging and to improve lifespan in mice [46,47,48]. Mitophagy is also reduced during aging, leading to mitochondrial dysfunction [47], whereas Parkin overexpression was shown to be sufficient to ameliorate cardiac aging in mice by rescuing autophagy [49]. The natural polyamine spermidine was also found to improve cardiac aging by boosting autophagy and mitophagy in mouse models of cardiac aging. Remarkably, epidemiological data revealed that dietary intake of polyamines correlated with the reduction of cardiovascular disease (CVD) and longevity in humans [47].

The role of autophagy in cardiac remodelling, hypertrophy and HF has been extensively dissected in mice subjected to pressure and volume overload. In models of pressure overload (PO), autophagy inhibition through cardiac Atg5 deletion contributed to cardiac dysfunction [37]. However, Beclin1 overexpression exacerbated cardiac dysfunction during PO [50]. Interestingly, heterozygous deletion of Beclin1 improved cardiac function [50], suggesting that physiological activation autophagy confers protection during PO, whereas its absence or its over-activation may be detrimental. Mitophagy seems to be the cargo-specific form of autophagy mainly involved during the process of cardiac remodeling. It is transiently activated during the initial phase of PO, thus representing an adaptive response. However, it rapidly decreases and mitochondrial dysfunction occurs. Reactivation of mitophagy through the synthetic peptide Tat-Beclin 1 rescued mitophagy and HF following PO [51]. In the same line of evidence, Parkin deficient mice subjected to myocardial infarction developed larger infarct size and reduced survival as compared to the wild type mice, because of an impaired activation of mitophagy in the border zone of the infarct. Notably, Oka et al. demonstrated that mitochondrial DNA that escapes from mitophagy during PO triggers myocardial inflammation and heart failure [52]. Although CMA has been reported to decline during aging or in metabolic disorders [53,54], there are no relevant studies about the mechanistic role of CMA in response to cardiac stress. However, since CMA is activated in response to oxidative stress or to hypoxic conditions, it will be interesting to assess the contribution of CMA during cardiac remodeling [55,56].

Autophagy was also found to be impaired in inherited cardiomyopathies, such as those associated with mutations in the αB-crystalline gene or in Danon’s disease [57,58,59]. In the first case, reactivation of autophagy through genetic overexpression or through physical exercise was found to improve cardiac function in mice harboring a mutation in αB-crystalline gene [60]. 

Furthermore, modulation of autophagy may be also a strategy to counteract the cardiotoxicity of doxorubicin and other anthracyclines, which typically evolves into HF. Several reports indicated that doxorubicin inhibits cardiac autophagy flux and that reactivation of autophagy antagonizes doxorubicin cardiotoxicity by enhancing the disposal of damaged mitochondria, which otherwise trigger severe derangement of cardiomyocytes [61,62]. Indeed, doxorubicin was shown to cause mitophagy impairment in the heart [49,63]. However, other studies showed that DOX also induces a detrimental autophagy in the heart [64,65]. In order to clarify this aspect, it has been demonstrated that slowing autophagy initiation improves autophagic flux and reduces DOX-induced cardiotoxicity, whereas increasing autophagosome formation exacerbates DOX damage of cardiomyocytes [61]. Another evidence indicates that restoration of TFEB is another potential strategy to counteract DOX-induced cardiac injury [63].

Overall, targeting autophagy represents a novel approach to prevent and treat HF. To date, several synthetic and natural activators of autophagy have been characterized and have shown to limit cardiac damage in different stress conditions [38,66]. However, given the dual role of autophagy in the heart, it should be important to maintain autophagy level in a physiological range. Moreover, recognition of autophagy in human samples should be implemented in order to translate the results obtained in experimental models to humans. 

## 4. Role of Inflammation in HF 

The association between inflammation and HF dates back to decades ago. However, the detailed inflammatory mechanisms in HF are still not entirely understood and their investigation represents a continuously expanding area of cardiovascular research. Paradigmatic examples are the recognition of elevated levels of C-reactive protein (CRP) and tumor necrosis factor-α (TNF-α) in HFrEF [67,68]. Based on this finding, a “cytokine hypothesis” has been postulated, according to which pro-inflammatory cytokines might exert harmful effects on cardiac function [69]. Despite compelling evidence of correlation between serum levels of inflammatory mediators and HF progression and prognosis [70], however, several phase III clinical trials testing anti-cytokine approaches have failed to show clear benefit or, in the case of anti-TNF-α, have even worsened clinical outcomes, challenging the notion that inflammation exclusively contributes to HF pathogenesis and may be a therapeutic target [71].

**Figure 2:**
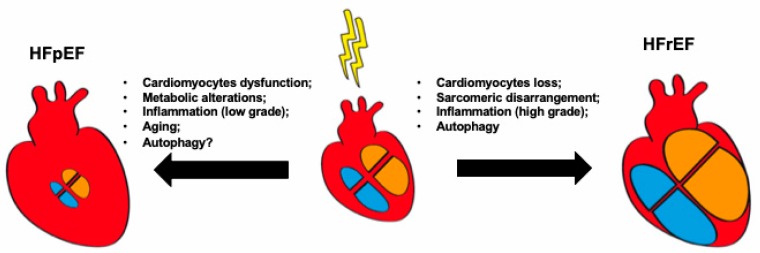
Key cellular alterations in heart failure with preserved ejection fraction (HFpEF) and heart failure with reduced ejection fraction (HFrEF).

Despite the differences between HFrEF and HFpEF, inflammation is a key feature of both [72]. Interestingly, distinct patterns of inflammation can be recognized in HFrEF vs. HFpEF. In post-ischemic HFrEF, inflammatory cells orchestrate cardiomyocyte remodeling and participate in scar formation and healing processes. Cell-, tissue-, and extracellular matrix (ECM)-derived products, collectively called damage-associated molecular patterns (DAMPs), trigger an immune response which is very similar to the one induced by infection [73]. This notion underlines the similarities between different types of inflammatory response and point to common main molecular regulators. Nonetheless, the role, extent and consequences of inflammatory mechanisms in HFrEF are only partially delineated. An example of how inflammatory mechanisms interact with other biological pathways in HF, is related to autophagy. Interestingly, the interaction between these two crucial biological processes (i.e., inflammation and autophagy) seems to be bi-directional. Pro-inflammatory cytokines and DAMPs release in HF can induce autophagy in the heart and in other organs [74,75]. At the same time autophagy is essential for regulating the inflammatory mechanisms [76]. As depicted in Figure 2, although autophagy has been implicated in the pathophysiology of HFrEF (discussed in the section above), very little is known about the role of autophagy in HFpEF. Therefore, the investigation of inflammatory regulation of autophagy, and vice versa, might represent a novel area of research in HF.

Whereas in HFrEF inflammation appears to be a deleterious consequence of myocardial injury, a causative relationship between inflammation and HF seems more evident in HFpEF (Figure 2). HFpEF typically arises from the presence of multiple comorbidities such as aging, hypertension, diabetes, and obesity, all of which have a common inflammatory ground. In HFpEF, endothelial inflammation precedes endothelial dysfunction, and is associated with impaired LV diastolic function and altered LV-arterial coupling. In this context, it has been proposed that coronary microvascular endothelial inflammation reduces endothelial nitric oxide (NO) availability with a subsequent decrease in cyclic guanosine monophosphate (cGMP) and protein kinase G (PKG) activity in cardiomyocytes [77]. Based on this, strategies aimed to increasing cGMP and NO availability have been tested in HFpEF. Unfortunately, these approaches have failed in providing substantial clinical benefit in HFpEF patients in large clinical trials. More recently, the attention has turned to the role of metabolic alterations supporting the inflammatory substrate observed in HFpEF [78,79]. The term “metabolic inflammation” or meta-inflammation has been used to describe the multitude of inflammatory events occurring in the setting of metabolic dysfunction [78,80]. For example, nutrient overload induces insulin resistance through the interaction with canonical inflammatory pathways such as the activation of IκB kinase-β (IKKβ) and JUN N-terminal kinase (JNK), leading to serine phosphorylation and activation of insulin receptor substrate (IRS) 1. Similarly, it is also known that inflammatory macrophages (i.e., M1) infiltrate adipose tissue in obesity and release cytokines that are responsible for the systemic pro-inflammatory state observed in this condition. In HFpEF, the meta-inflammatory activation of inducible NO synthase (Pantos, #84), which has been demonstrated in both experimental models of HFpEF and human hearts from HFpEF patients, triggers cardiomyocyte dysfunction and has been shown to be a key mechanistic driver of this syndrome [78]. Although caution is warranted, iNOS and other pro-inflammatory molecules (e.g., IL-1) [81] have been proven to be potentially valuable inflammatory therapeutic targets for HFpEF. In conclusion, further research dissecting the inflammatory pathways active in HF is essential to solve the complexity of inflammation in HF and possibly discover novel therapeutic interventions.

## 5. Role of Gut Microbiota in HF

**Figure 3:**
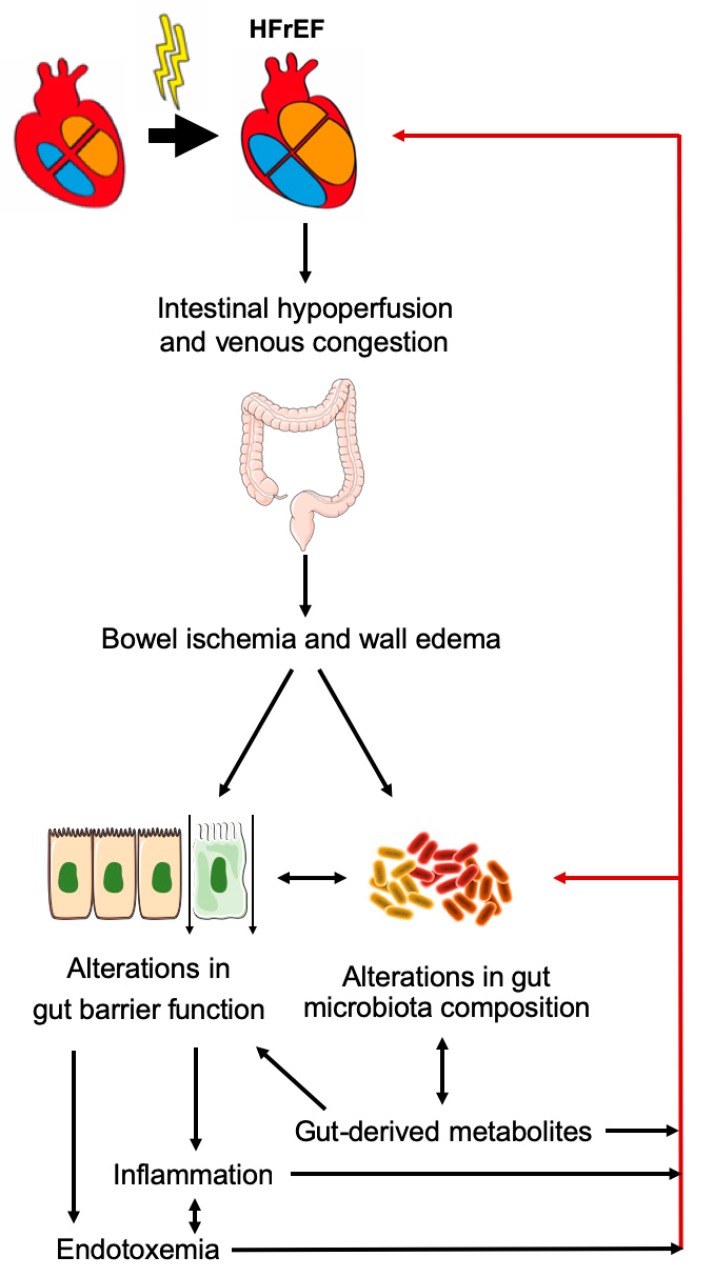
Gut-heart crosstalk in heart failure with reduced ejection fraction (HFrEF).

Alterations in gut barrier function and microbiota composition are emerging hallmarks of HF [82,83]. In patients with HFrEF, intestinal hypoperfusion and venous congestion determine bowel ischemia and wall edema, affecting bacterial growth and increasing intestinal permeability [84,85]. These perturbations promote a persistent low-grade inflammation in the gut, intestinal epithelial disruption and risk of endotoxemia [86]. At the same time, systemic increase in inflammatory cytokines aggravates gut permeability, promoting lipopolysaccharide (LPS) translocation and systemic inflammation [87]. Alterations in gut microbiota pathways and changes in circulating levels of metabolites, including those derived from dietary nutrients, can exert paracrine or endocrine effects and modulate HF development or progression. Collectively, these changes can aggravate HF in a vicious, self-amplifying cycle (Figure 3).

Alterations in gut permeability, changes in diversity and composition of gut microbiome and elevated circulating levels of LPS have been already identified in patients with HF, triggering a state of chronic inflammation [84,87,88]. Several relatively small studies in patients have attempted to characterize HF gut microbiota profile, even if inconsistencies and discrepancies have also been outlined [82]. Since gut microbial transplantation has been already proven to provide diseases susceptibility or protection, a deeper understanding of gut microbiota composition and mechanistic studies to determine whether and how gut microbiota contributes to HF will be needed [89,90].

Identification of mediators of gut-heart crosstalk, and of strategies to modulate such communications, might also be crucial to find novel diagnostic, prognostic or therapeutic targets for HF patients. Gut microbiome-derived metabolites such as trimethylamine N-oxide (TMAO), short-chain fatty acids (SCFA) or secondary bile acids (BA) [5] have been shown to play critical roles in cardiac health and disease [91,92]. Although elevated TMAO levels have been identified in patients with HF, increasing long-term mortality risk independently of traditional risk factors [93], the mechanisms underlying such detrimental effects are still unclear. Similarly, SCFA seem to be mediators of beneficial effects elicited by the gut microbiome in several CVD [94]. SCFA bind several transmembrane receptors, mediating effects on blood pressure, glucose homeostasis, appetite, obesity [94]. In particular, butyrate has long been recognized important to maintain gut barrier function and to exert beneficial effects when administered exogenously in several experimental models of CVD [95,96]. Recently, propionate has also been shown to significantly attenuate cardiac hypertrophy, fibrosis, vascular dysfunction, and hypertension in two different mouse models of hypertensive cardiovascular damage [97], even if its precise role in HF remains largely undetermined. Gut microbiota is also involved in the secondary conversion of BA synthesized by the liver, modulating gut microbial composition, activating innate immune response genes in the small intestine [98], affecting in turn weight management and obesity, insulin secretion and sensitivity, hepatic lipid metabolism and hypertension [98].

Several strategies have been hypothesized to interrupt the gut-heart axis in HF, including targeting gut microbiota composition, microbial metabolic pathways and products or gut function and permeability. Specific dietary recommendations, the use of prebiotics, probiotics, or antibiotics might transform gut microbial community and function. Although fecal microbiota transplantation from healthy donors has been proven effective for severe cases of *Clostridium difficile* infection, it might potentially increase the risk of transferring subclinical disease phenotypes from apparently healthy donors. Thus, other strategies including the administration of single microbial species, cocktails of defined species, or agents that target specific microbiota-derived molecules might be tested. Furthermore, the use of non-lethal microbial enzyme inhibitors might be less likely to promote microbial resistance. To achieve changes in microbial metabolites, it is possible to speculate that sequestrant resins capable of intercepting specific microbial metabolites might prevent or reduce absorption by the host [87]. Importantly, the role of host genetic profile, sex, comorbidities, and associated comedications, the effects of antibiotics, probiotics and diet regimens is far from being elucidated and will deserve careful evaluation in future studies. Finally, the role of microbe–microbe interactions might represent an additional knowledge gap and will require additional investigation.

## 6. New Modalities for HF Drug Delivery

The latest scientific discoveries in the field have led to a significant incremental advance toward potential new cardiovascular therapies to patients. However, these numbers did not proportionally translate into effective EMA and FDA approved drugs. From one side, this relies on the prohibitively expensive trials and drop in investments toward other therapeutic field such as oncology and immune therapy. On the other side, the lack for an efficient, safe and selective drug administration system to the heart that avoids side effects to non-diseased organs [99]. In this context, the emerging biomedical field of “nanotechnology for controlled drug delivery” may provide valid tools to effectively circumvent the current limits [100].

Nanotechnology relies on the application of nanocarriers (NCs) that, being either viral- (vNC) or non-viral (nvNC), represent any particulate with at least one dimension between the range of 1–100 nm [100]. vNCs in CVD mainly belongs to the AAV (Adeno-Associated-Virus) vector type. AAVs are small and non-enveloped icosahedral capsids containing a single-stranded DNA engineered to contain the therapeutic gene of interest [101]. Based on the serotype (12 strains so far identified) and the tissue promoter specificity, AAV vectors can be used to transduce genetic material in a broad range of cell type and organs. For CVD gene transfer, serotypes AAV1, AAV6, and AAV9 have emerged as the most promising ones and several pre-clinical works are currently translating toward Ph1 studies (e.g., Audentes Therapeutics with the AAV-CASQ2 gene therapy for the long-term treatment of Catecholaminergic Polymorphic Ventricular Tachycardia). Unfortunately, challenges like transduction to off-target organs, and AAV neutralization caused by pre-existing immunity still hamper the great potential for clinical application of such viral-based NCs. Failure of the CUPID2/AAV-SERCA trial (NCT01643330) to reach the final phase for the pharmaceutical approval is an example [102].

In contrast to vNCs, non-viral vectors possess additional features suitable for drug delivery purpose in terms of wider type of payload (i.e., from nucleic acid to amino acid, to synthetic hydrophobic and hydrophilic molecules), increased bioavailability, protection against drug degradation, higher efficiency, simple production method, less immunogenicity and low cost [103,104]. Furthermore, in addition to therapeutic purposes, nvNCs might be engineered as imaging agents (e.g., ferumoxytol for magnetic resonance imaging of myocardial infarction, NCT01323296). Depending on their composition, nvNCs can be classified as organic (lipid complexes, dendrimer, polymers), or inorganic (calcium phosphate, silica, gold and magnetic nanoparticles, quantum dots, carbon nanotubes) [105]. In addition, despite being generated just as a plain drug carrier, nvNCs, based on the intrinsic chemical and physical characteristics, may play as a stimuli-responsive element providing an additional strategy for enhanced and controlled guidance. In this case, enhanced drug release at the target site can be achieved by modification of external condition (e.g. pH, electromagnetic field) [106]. Based on the type of nanomaterial together with the preparation technique, nvNCs might differ in terms of size, shape, surface charge and functionalization thereby affecting their biodistribution and activity [100,107]. A size ranging between 50 and 100 nm has been shown with an extended half-life in the blood circulation and an enhanced probability to extravasate the stressed vessels (enhanced permeation and retention, EPR effect) toward the cardiac tissue. For example, the alteration of vascular permeability at the ischemic site post myocardial infarction facilitates the translocation and accumulation of nvNCs toward myocardial cells. On the contrary, NCs smaller than 30 nm are easily excreted through the kidney [108] while NCs larger than 200 nm tend to be sequestered by macrophages, targeted to the reticuloendothelial system, and faster removed from the circulation [109,110,111]. Thus, in combination also with a specific route of administration (i.e., direct injection, inhalation or oral intake), the in vivo biodistribution of specific nvNCs is affected. In the case of inhalable calcium phosphate nanoparticles (<100 nm), nvNCs cross translocate the alveoli and via the lung-to-heart bloodstream fast reach the heart [112,113]. Finally, a further key role is played by the charge and functionalization of nvNC surface. In fact, positive, negative, or even neutral a charge, may affect nvNC stability, distribution in the blood, and safe interaction with the targeted cells. Within the heart, polarized cells like cardiomyocytes emerged to be more attractable and compatible with negative charged nanoparticle via the formation of life-compatible nanopores for their internalization [112,113]. Surface functionalization is extensively explored for a further advancement toward a more selective delivery of the therapeutic cargo toward cell-specific tissue districts, minimizing or avoiding the toxic off-targets effects on healthy cells or tissues [107,114]. Despite no clinical trials are yet available, many cell-specific (or -enriched) targeting ligands like peptides, antibodies, and more recently aptamers, are currently explored. In conclusion, nanotechnology is a promising tool to deliver drugs to the heart and, thereby, treat HF. 

## 7. Conclusions

HF remains inadequately managed in spite of a variety of medications and interventional procedures being available, with ensuing substantial morbidity and mortality. Efforts are being pushed to optimize the use of established and recently approved therapies [2,115], but it appears unlikely that they will be sufficient. Hence, innovative strategies are required for the effective targeting of the main cause of the disease rather than ameliorating the symptoms. The combination of nanotechnological approaches for further precise cardiac delivery of either novel or clinical available drugs together with additional biomarkers and imaging technologies might fulfil this critical need. The knowledge and technologies are provided by basic science, which therefore represents an invaluable resource to achieve better diagnosis, prognostic stratification and treatment of HF, and finally conquer it.

## Figures and Tables

**Table 1:** Genes Associated with Inherited Cardiomyopathies.

Gene	Gene Name	DCM	HCM	ACM	Inheritance
*ACTC*	α-Cardiac actin	Χ	Χ		AD
*ACTN2*	α-Actinin2	Χ	Χ		AD
*BAG3*	BCL2-associated athanogene 3	Χ			AD
*CDH2*	Cadherin 2			Χ	AD
*CSRP3*	Cysteine and glycine-rich protein 3	Χ	Χ		AD
*DES*	Desmin	Χ		Χ	AD, AR
*DSC2*	Desmocollin 2	Χ		Χ	AD
*DSG2*	Desmoglin 2	Χ		Χ	AD
*DSP*	Desmoplakin	Χ		Χ	AD, AR
*FLNC*	Filamin C	Χ	Χ	Χ	AD
*GLA*	α-Galactosidase		Χ		XL
*JUP*	Junctional plakoglobin			Χ	AD, AR
*LDB3*	LIM-domain binding 3	Χ			AD
*LMNA*	Lamin A/C	Χ		Χ	AD
*MYBPC3*	Myosin binding protein C	Χ	Χ		AD
*MYH7*	β-Myosin heavy chain 7	Χ	Χ		AD
*MYL2*	Myosin regulatory light chain 2		Χ		AD
*MYL3*	Myosin light chain 3		Χ		AD
*PKP2*	Plakophilin 2			Χ	AD
*PLN*	Phospholamban	Χ	Χ		AD
*PRKAG2*	AMP=activated protein kinase, γ2, noncatalytic		Χ		AD
*RBM20*	RNA binding motif protein 20	Χ			AD
*RYR2*	Ryanodine receptor 2			Χ	AD
*SCN5A*	Voltage-gated sodium channel, α subunit	Χ			AD
*TMEM43*	Transmembrane 43			Χ	AD
*TNNC1*	Cardiac troponin C, type 1	Χ	Χ		AD
*TNNI3*	Cardiac troponin I, type 3	Χ	Χ		AD
*TNNT2*	Cardiac troponin T, type 2	Χ	Χ		AD
*TPM1*	α-Tropomyosin 1	Χ	Χ		AD
*TTN*	Titin	Χ	Χ	Χ	AD
*TTR*	Transthyretin	Χ	Χ		AD

AD: autosomal dominant; AR: autosomal recessive; XL: X-linked.
